# Is there echinococcosis in West Africa? A refugee from Niger with a liver cyst

**DOI:** 10.1186/s13071-017-2169-6

**Published:** 2017-05-11

**Authors:** Andrea Angheben, Mara Mariconti, Monica Degani, Maria Gobbo, Loredana Palvarini, Federico Gobbi, Enrico Brunetti, Francesca Tamarozzi

**Affiliations:** 1 Centre for Tropical Diseases, Hospital Sacro Cuore, Negrar, Verona, Italy; 2 Division of Infectious and Tropical Diseases, San Matteo Hospital Foundation, Pavia, Italy; 3 WHO Collaborating Centre for Clinical Management of Cystic Echinococcosis, Pavia, Italy; 4 Service of Epidemiology and Laboratory for Tropical Diseases, Hospital Sacro Cuore, Negrar, Verona, Italy; 5 Infectious Diseases, Ospedale Carlo Poma, Mantova, Italy; 6 Department of Clinical Surgical Diagnostic and Paediatric Sciences, University of Pavia, Pavia, Italy

**Keywords:** Cystic echinococcosis, West Africa, Migration

## Abstract

**Background:**

Italy is presently facing an increase in immigration from sub-Saharan Africa through the Mediterranean Sea. Case reports of human cystic echinococcosis (CE) have been reported from most sub-Saharan countries. Therefore, an increase in the number of patients with CE coming from these areas in the Italian and European centers for infectious diseases is expected. Unfortunately, the epidemiology of CE in sub-Saharan countries is poorly known, which makes clinical suspicion and diagnosis of such infection difficult in patients coming from these areas.

**Results:**

Here we report a case of hepatic CE in a patient from Niger who arrived in Italy through Libya and visited in a Tropical Medicine referral center in Northern Italy. The parasite was identified molecularly as the G6 “camel” strain of *Echinococcus granulosus* (*E. canadensis*). The diagnosis and management of a chronic and clinically complex infection like CE in such situation is difficult. Only 40 cases of CE from Niger have been reported; of these, 75% had extra-hepatic localization. To our knowledge, no strain characterization of human isolates from Niger has been reported so far. The CE cyst of the patient was in CE3a stage, indicating active transmission from the area in which the patient came. However, prevalence data from Niger, and from any other country in West Africa, are almost inexistent.

**Conclusions:**

We argue that population epidemiology surveys with ultrasound are warranted in Sahelian countries, including Niger. These studies could improve the knowledge of CE epidemiology, provide health authorities with important information for public health interventions targeting this zoonosis, and shed light on any difference between tissue tropism and clinical manifestations caused by the different *E. granulosus* strains.

## Background

Human cystic echinococcosis (CE) is a neglected zoonotic parasitic disease caused by the larval stage of the dog tapeworm *Echinococcus granulosus* (*sensu lato*) species complex, with a biological predator-prey cycle occurring naturally between dogs and ungulates. The transmission cycle occurs worldwide, but the infection is most prevalent in pastoral communities, causing considerable socioeconomic impact [1]. Humans are occasional, “dead-end” intermediate hosts, where the parasite develops in its larval stage as fluid-filled cysts (commonly called hydatid cysts) in organs and tissues, most commonly in the liver, followed by the lungs. Infection may cause a wide range of clinical presentations, from asymptomatic to life-threatening [2]. Most hepatic infections are pauci/asymptomatic [3] and are detected *via* ultrasound (US) examination during investigation of right upper quadrant abdominal pain, the most common reported symptom, or by chance during an unrelated US exam.

Italy is endemic for CE, but data from the World Health Organization Collaborating Centre in Pavia collected in the Italian (now European) Register for CE indicate that 38% of patients with CE visited in this centre are foreign born [4, 5]. Italy is presently facing an increase in immigration from sub-Saharan Africa. Data from the United Nations High Commissioner for Refugees indicate that in 2013 about 43,000 people arrived in Italy by sea through the Mediterranean route. This figure more than quadrupled in 2016, when over 180,000 people reached Italy through the Mediterranean Sea, departing from Libya in 90% of the cases. Those arriving by sea mainly originate from sub-Saharan Africa (over 40%). In 2016, 21% of all arrivals originated from Nigeria, followed by Eritrea (11%), Guinea, Côte d’Ivoire and the Gambia (7% each) (https://data.unhcr.org/mediterranean/country.php?id=105, accessed on January 31st 2017). Case reports of human CE have been reported from most sub-Saharan countries [6–9]. Therefore, an increased number of patients with CE coming from these areas to be visited in Italian and European centers for infectious diseases is expected. CE is a chronic and clinically complex disease, requiring prolonged medical care and follow-up, with substantial costs [10, 11]. Unfortunately, the epidemiology of CE in sub-Saharan countries is poorly known, which, adding to the general neglect of CE outside referral centers, makes clinical suspicion and diagnosis of such infection even more difficult in patients coming from these areas.

Here we report a case of hepatic CE in a patient from Niger who was admitted in a Tropical Medicine referral center in Northern Italy, and discuss the epidemiological implications deriving from this occurrence.

## Methods

Parasite genotyping was performed using multiple primers and confirmed by sequencing. Briefly, genomic DNA was extracted from the cyst fluid sediment using the DNeasy Blood & Tissue kit (Qiagen, Valencia, CA, USA), according to the manufacturer’s instructions. Fragments of cytochrome *c* oxidase subunit 1 (*cox*1) of *E. granulosus* (*sensu lato*) [12], *E. granulosus* (*sensu stricto*) G1-G3 and *E. ortleppi* G5 [13]; elongation factor 1 alpha (*ef1a*) of *E. granulosus *(*s.s*.) G1-G3; calreticulin (*cal*) of *E. equinus* G4; DNA polymerase delta (*pold*) of *E. canadensis* G6-G7; and ezrin-radixin-moesin-like protein (*elp*) of *E. canadensis* G8-G9-G10 (all [13]) were amplified by PCR, using published specific primers and thermic profiles (*cox*1 [12]; *cox*1, *rpb2*, *ef1a*, *cal*, *pold* and *elp* [13]). After gel electrophoresis, PCR products were purified with the Wizard DNA Clean-Up System (Promega, Madison, WI, USA), and sequenced (PRIMM, Milan, Italy). Analysis of nucleotide sequence data was performed with BLAST algorithms and databases from the National Center for Biotechnology (http://www.ncbi.nlm.nih.gov). The newly generated sequences where submitted to the GenBank database under accession number KY996491.

## Results

A 26-year-old man from Niger was admitted to the Centre for Tropical Diseases, Hospital Sacro Cuore, Negrar, Verona, Italy, in October 2012 for recurrent upper right quadrant pain. He had lived in Niger until 2010, when he moved to Libya, where he remained from August 2010 until August 2011 before moving to Italy. He reported working as a butcher in his home country and in Libya. His remote clinical history was unremarkable, with just a reported episode of hospitalization when he was a 12 year-old child due to an accident resulting in a thoracic wound. No documentation or report of a previous abdominal imaging investigation was given by the patient. A first diagnostic workup carried out in the first health-screening centre in Mantova (Italy) evidenced chronic HBV-HDV hepatitis. Complete blood count on admission showed hypereosinophilia (6.5%), which had also been reported in previous analyses performed in Mantova. An abdominal US scan showed a round focal lesion of 9 cm in diameter in VI-VII segments, with anechoic content and wavy linear wall echoes consistent with an echinococcal cyst in CE3a stage, according to the WHO-IWGE (Informal Working Group on Echinococcosis) international classification [12]. Serology for echinococcosis was positive on ELISA (34.04 Index positive > 11; DRG Instruments GmbH, DRG International, Marburg, Germany) but not on IHA (Cellognost-Echinococcosis, Dade Behring, Marburg, Germany).

Due to the size of the cyst, a percutaneous treatment approach was decided, according to the WHO-IWGE Expert Consensus stage-specific approach to liver CE [14]. Following a week of oral albendazole 400 mg BID, the cyst was punctured in local anaesthesia under US guidance and a pigtail catheter was placed, resulting in 520 ml of cloudy fluid drainage. Microscopic examination of the fluid showed non-viable protoscoleces and hooklets. The catheter was removed after 4 days and a control US scan performed 2 days later showed a little reduction in cyst diameter (8.5 cm) but with increase in its solid content. The patient was discharged 4 days after catheter removal, in a good condition and subjectively without abdominal pain. He was prescribed a 3 months therapy with albendazole and follow-up in Mantova, but unfortunately the patient did not show up and was lost to follow-up. Parasite genotyping obtained through cytochrome *c* oxidase subunit 1 (*cox*1) and DNA polymerase delta (*pold*) nucleotide sequencing showed maximum homology (> 99%) with the G6/G7 genotype (*E. canadensis*) sequences registered in the GenBank database.

## Discussion

Although it is not possible to completely exclude the hypothesis that our patient acquired infection during his stay in Libya, also endemic for CE, the size of the cyst and the relatively short time elapsed between arrival in Libya and diagnosis of CE in Italy (2 years) strongly favour the acquisition of infection in Niger. Indeed, although the natural history of CE is still poorly documented, it has been estimated that more than 90% of cysts grow slowly, less than 1.5 cm/year [15].

Niger is a vast landlocked country with an estimated population of 19.9 million people, most of whom live along a narrow band of arable land on the country’s southern border. The economy is dominated by agricultural activity. Life expectancy at birth is 61 years. Niger is an extremely poor country, with a *per capita* GNI (Gross National Income) of US$ 390; 48.9% of the population live below the national poverty lines (World Bank http://web.worldbank.org/WBSITE/EXTERNAL/COUNTRIES/AFRICAEXT/NIGEREXTN/0,,menuPK:382456~pagePK:141159~piPK:141110~theSitePK:382450,00.html, accessed on January 30th, 2017). Italy is among the ten most frequent countries of destination of emigrants from Niger, that also include eight West African countries and Germany; however few of them seek asylum in Italy (http://publications.iom.int/system/files/pdf/niger_profile_2009.pdf, accessed on February 2nd, 2017). Indeed, little over 1200 people from Niger were officially residing in Italy in 2015 (http://www.comuni-italiani.it/statistiche/stranieri/rn.html, accessed on February 2nd, 2017). The diagnosis and management of a chronic and clinically complex infection like CE in such situation is difficult. CE has a broad range of clinical manifestations, from asymptomatic to life-threatening complications, and of differential diagnoses. Therefore, the final diagnosis often occurs after referral to specialized centres. Its clinical management is often expensive, and clinical decision-making is often complex as it is based on cyst location, stage, size, complications, and other clinical variables [14]. Unfortunately this approach is poorly known outside referral centres [16]. After diagnosis and treatment, the patient requires long follow-up (years), to detect cyst reactivation or late complications as early as possible. Loss to follow-up early after diagnosis or treatment, as it often occurs in the case of asylum seekers moving continuously, may pose serious risks for patients’ health.

There are very few reports of human CE from Niger. A total of 40 cases of CE in patients who never left Niger have been published in PubMed (MEDLINE), all dating between 1985 and 1991 [17–21]. Among these, in only 10 cases (25%) CE cysts were located in the liver. Develoux and colleagues [21] advanced the hypothesis that the apparent low frequency of CE in the Sahelian countries of West Africa could be due to the predominance of the *E. granulosus* camel strain, with a cycle maintained mostly between camels and dogs, rather than sheep and dogs, as dogs are not commonly used as sheep guards in these areas. These authors [21] report that none of 4795 sheep slaughtered in three abattoirs of Niger and none of 76 dogs necropsied in Niamey were found to be infected by *E. granulosus*. Very low prevalence of dog and sheep infection are also reported from neighbouring countries (North-East Nigeria: 1.2% for dogs, 7% for sheep; Chad: 3.4% for dogs, 0.2% for sheep) [7, 21], compared to figures reported in African endemic countries. On the contrary, camels are more commonly infected, with reported prevalence ranging from 3% to over 20% in Niger and up to 70% in Nigeria [7, 21], although these figures may be partly biased due to the different age of slaughter of sheep and camels.

The G6 “camel” strain of *E. granulosus *(*s.l*.) , also defined as *E. canadensis*, is the second most common genotype causing human infection, after the G1-G3 “sheep” strain, i.e. *E. granulosus* (*s.s*.) [6]. In particular, the G6 strain has been found in 12.2% of typified human isolates [6]. Interestingly, all reports of typified human CE cases from Sahel were of the G6 strain [6, 7, 22]. To our knowledge, no strain characterization of human isolates from Niger has ever been reported.

There is virtually no data on the different clinical manifestations, if any, caused by the different *E. granulosus *(*s.l*.) genotypes. It has been proposed that the G6 strain may have a different tropism from the G1 strain, with a predominance of extra-hepatic “rare” localizations [22]. However, this is difficult to affirm with current available data, based on case reports from hospitalized cases, which are highly biased in terms of clinical representation and publication of results. Indeed, available data show that the liver is the prevalent localization of all three genotypes most commonly affecting humans, including G6: G1-G3 (73%), G6 (54%) and G7 (98%) [6].

The CE cyst of our patient was in CE3a stage. This is a transitional stage, from early active cyst (CE1) to, presumably, spontaneous inactivation [14]. It has been shown using 1H-MRI spectroscopy that about half of CE3a cysts are biologically active, and half inactive, in the absence of previous therapy [23]. This latter was indeed the case of the cyst of our patient. In any case, CE3a is an early stage in the natural evolution of CE cyst, indicating active transmission in the area from which the patient came. However, prevalence data from Niger, and from any other country in West Africa, are almost inexistent.

## Conclusions

As CE has important health and socioeconomic impact on the rural populations affected, we believe that population epidemiology surveys with ultrasound are warranted in Sahelian countries, including Niger, where pastoralism is largely practiced. These studies could improve our knowledge of CE epidemiology, provide health authorities with important information for public health interventions targeting this zoonosis, and shed light on any difference between tissue tropism and clinical manifestations caused by the different *E. granulosus* strains.

## Abbreviations

CE: Cystic echinococcosis
GNI: Gross National Income
US: Ultrasound
WHO-IWGE: World Health Organization Informal Working Group of Echinococcosis
